# Metamaterials and imaging

**DOI:** 10.1186/s40580-015-0053-7

**Published:** 2015-11-09

**Authors:** Minkyung Kim, Junsuk Rho

**Affiliations:** 1 Department of Mechanical Engineering, Pohang University of Science and Technology (POSTECH), Pohang, 790-784 Republic of Korea; 2 Department of Chemical Engineering, Pohang University of Science and Technology (POSTECH), Pohang, 790-784 Republic of Korea

**Keywords:** Metamaterials, Plasmonics, Optical microscope, Diffraction limit, Super-resolution imaging

## Abstract

Resolution of the conventional lens is limited to half the wavelength of the light source by diffraction. In the conventional optical system, evanescent waves, which carry sub-diffraction spatial information, has exponentially decaying amplitude and therefore cannot reach to the image plane. New optical materials called metamaterials have provided new ways to overcome diffraction limit in imaging by controlling the evanescent waves. Such extraordinary electromagnetic properties can be achieved and controlled through arranging nanoscale building blocks appropriately. Here, we review metamaterial-based lenses which offer the new types of imaging components and functions. Perfect lens, superlenses, hyperlenses, metalenses, flat lenses based on metasurfaces, and non-optical lenses including acoustic hyperlens are described. Not all of them offer sub-diffraction imaging, but they provide new imaging mechanisms by controlling and manipulating the path of light. The underlying physics, design principles, recent advances, major limitations and challenges for the practical applications are discussed in this review.

## Introduction

Curiosity and desire to see microscopic world have leaded the invention of the optical microscope. Thanks to the optical microscope, the small structures that had been invisible to the naked eyes could be seen. Modern biology and medical science where large portions are based on the observation of micro-objects such as cells and bacteria have been developed with the advance in the optical microscope. With fluorescent materials, optical microscopes became more useful and essential for the decades, but there is still a fundamental question how to achieve the spatial resolution below diffraction limit.

In 1870s, Abbe discovered that when an object is observed by optical devices such as microscopes or telescopes, the features smaller than half of the wavelength of light are not resolvable because of diffraction phenomena [1]. Due to diffraction limit, resolution achievable by using conventional optical microscope is intrinsically limited to approximately two hundred nanometers in visible light. Realizing microscopes with higher resolution have been active research area. At 1930s, microscopes using electrons such as scanning electron microscope (SEM) [2] and transmission electron microscope (TEM) [3] were invented. Those two microscopes have much higher resolutions of tens of nanometers compared to the typical optical microscope due to the shorter de Broglie wavelength of electrons. However, since studying living specimens is not compatible with the electrons in general, the electron microscopes are inadequate for the biological applications although they are the most common imaging tools to visualize the nanoscale structures. Around the same time, near-field scanning optical microscope (NSOM) in which evanescent waves are obtained by putting detector very close to an object was proposed [4, 5]. Lateral resolution of 20 nm and vertical resolution of 2–5 nm have been achieved [6, 7]. However, long scanning time and tricky conditions such as very short distance between the detector and the object were the obstacles for the practical imaging applications. At 1980s, the spatial resolution of order of few nanometers was realized by atomic force microscopy (AFM) [8, 9]. AFM has many advantages including capability of 3D imaging and compatibility in liquid environment, but very small single scanning size and ow scanning speed raised problems. The biggest limitation of NSOM and AFM is that both do topography imaging which examines only the surface in the near-field.

To overcome such limitations, far-field microscopy with super-resolution and bio-compatibility have been successfully developed and widely used. Localization microscopy techniques such as stochastic optical reconstruction microscopy (STORM) [10] and photo activated localization microscopy (PALM) [11, 12] using photoswitchable fluorophores were proposed. And, as non-localization approaches, stimulated emission depletion (STED) [13] microscopy using two laser pulses to achieve 3D/multi-color/video-rate imaging accomplished 6 nm spatial resolution [14]. Saturated structured-illumination microscopy (SSIM) [15] using nonlinear dependence of fluorophores and structured illumination microscopy (SIM) [16] using grid patterned incident light have been also developed. However, for the microscopes using laser, samples can be damaged by high-intensity pulse of the laser, and for the microscopes based on fluorescent materials, resolutions are limited by the labeling density and the size of the fluorescent labels [17]. In addition to the development of such advanced imaging methods, various algorithms and techniques to realize super-resolution have been also studied [18].

In this review, we will describe the new types of imaging approaches based on new optical materials called metamaterials which can provide the direct control and manipulation of electromagnetic properties to overcome diffraction limit. Metamaterial-based imaging composed with the special lenses, for example, but not limited to, superlenses, hyperlenses, metalenses, and non-optical lenses such as acoustic hyperlens will be reviewed, and the underlying physics, design principles, recent advances, major limitations and challenges for the practical applications will be discussed in each section. Since the lenses are named by proposers, not by social protocols, the boundaries of the divided groups may be ambiguous.

## Review

### To overcome diffraction limit

The reason why the conventional lenses have suffered from diffraction limit is that waves with high transverse wavevectors (evanescent waves) decay exponentially during propagation since most of the naturally available materials have both positive permittivity and permeability for all directions.

1 $$ \frac{{k_{\perp}}^2}{\varepsilon_{\parallel }}+\frac{{k_{\parallel}}^2}{\varepsilon_{\perp }}={\left(\frac{\omega }{c}\right)}^2 $$

**Fig. 1:**
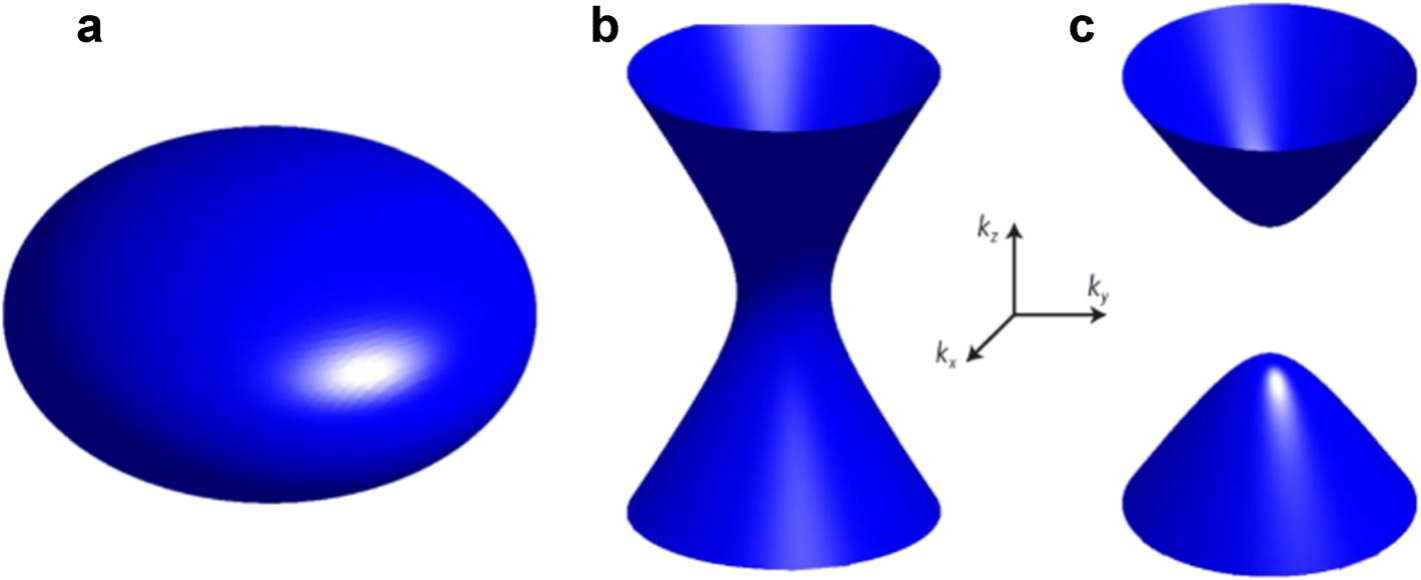
Isofrequency surface. **a** Elliptical isofrequency surface. **b** and **c** Hyperbolic isofrequency surface with (**b**) ***ε***_⊥_ = ***ε***_***x***_ = ***ε***_***y***_ < 0, ***ε***_∥_ = ***ε***_***z***_ > 0, and (**c**) ***ε***_⊥_ = ***ε***_***x***_ = ***ε***_***y***_ > 0, ***ε***_∥_ = ***ε***_***z***_ < 0

Here, equation (1) is the dispersion relation of transverse magnetic (TM) waves in a uniaxial medium derived from Maxwell’s equations where the subscripts ⊥ and ∥ denote components perpendicular and parallel to the propagating direction, respectively, k is the wavevector, ε is the relative permittivity, n is the refractive index of the medium, ω is the angular frequency, and c is the speed of light in vacuum. From the dispersion relation, natural materials have closed isofrequency surface- ellipsoid or sphere, as shown in Fig. 1 (a). It means that for the waves with all real components wavevector, wavevector components of transverse direction (*k*_⊥_) are bounded. Evanescent waves have higher *k*_⊥_ than the bounded value, and therefore, they carry sub-diffraction information. However, since the propagating component (*k*_∥_) is imaginary from the dispersion relation, the amplitude will exponentially decay and the sub-diffraction information cannot reach to the image plane.

Meanwhile, materials with hyperboloid isofrequency surface, as shown in Fig. 1 (b) and (c), can deal with the evanescent waves since *k*_⊥_ can have unbounded value. More generally, if there exists a lens in which the evanescent waves can be transferred from the object plane to the image plane without exponential decay, diffraction limit could be overcome. In addition, if impedance of the lens is matched to one of air so that all the incident light are transmitted through the lens without any reflection, every beam from the object plane will reach the image plane. It indicates that theoretically perfect lens with infinite resolution can be realized. Such a perfect lens has been proposed and studied by metamaterials.

Metamaterials are artificially structured materials composed of the building blocks of which size and spacing of atoms are at deep sub-wavelength scale. Their extraordinary electromagnetic responses are determined by the size, shape and arrangement of the unit cells, not by chemistry. By arranging those building blocks properly, metamaterials exhibit fascinating optical properties that do not exist in nature such as negative refractive index, invisibility cloak, artificial chirality, superlensing and hyperbolic dispersion relation.

Negative index materials (NIMs), one of the most interesting applications of metamaterials, were first proposed by Veselago in 1960s. He predicted that a hypothetic material where both ε and μ are negative has negative refractive index. At the interface of positive index medium and negative index medium, light will be negatively refracted and the energy and phase will flow in the opposite direction in the medium of negative index. It was also noted that negative refraction causes many other unusual phenomena such as reversed Doppler/Cherenkov effect [19].

**Fig. 2:**
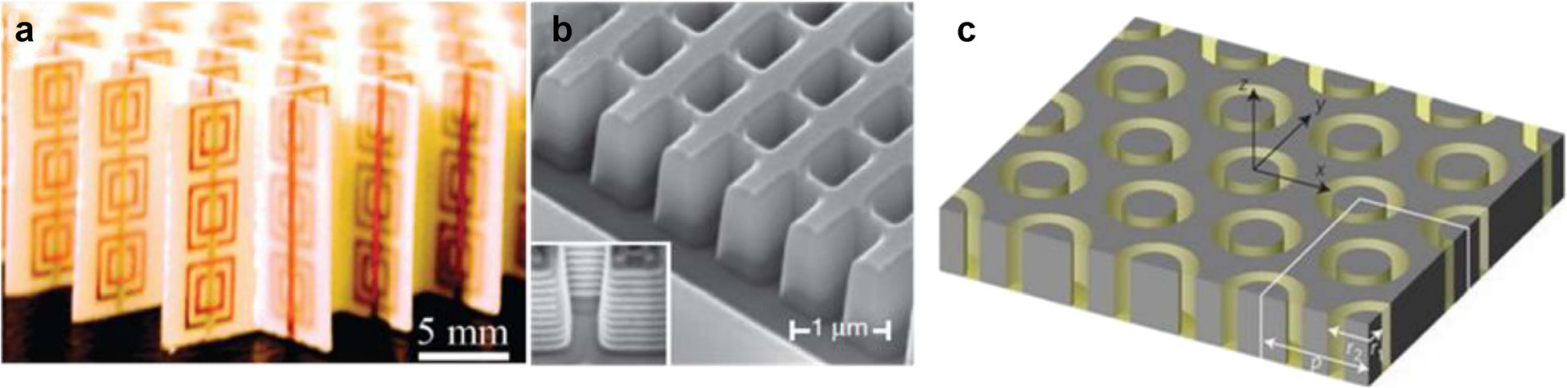
Examples of negative index materials. **a** The first demonstration of NIM by SRR array with metallic wires in microwave. [21] **b** SEM image of a fishnet structure which functions as a near-infrared (NIR) frequencies operating NIM [26]. **c** Polarization-independent NIM based on a single-layer coaxial waveguide structure [34]

However, there had not been many further studies since there was no such a material in nature until the first NIM was realized experimentally. It was proved that split-ring resonator (SRR) structure with metallic wires exhibits negative ε and μ at microwave frequency by Smith [20, 21]. After then, other SRR structures showing negative refractive index at microwave frequency have been realized by other groups [22–24]. Further, different designs of NIM in various operating frequencies such as printed metallic strip [25], fishnet structure [26], and chiral resonators [27, 28] have been also proposed. The early demonstrations of such properties of the metamaterials had some limitations. For example, their exotic properties exist along only one direction or under special conditions such as specific polarization, normal-incident condition, the limited wavelength range above microwave, and short bandwidth [29–32]. However, recently, many efforts to design metamaterials that are free from those constraints have been actively made and demonstrated in many ways. The examples are as following, but not limited to, orientation-independent bulk NIM for TM wave [33], isotropic and polarization-independent bulk NIM [34], and bulk NIM at visible frequency [35, 36]. Representative examples of NIM are shown in Fig. 2.

Thanks to such extraordinary optical properties, metamaterials have shown great potentials in many applications including waveguide [34, 35], artificial magnetism [37], spontaneous emission enhancement [38], and transformation optics [39, 40], and one of the most exciting applications is the diffraction-free imaging which is also called sub-diffraction or super-resolution imaging.

### Perfect lens from negative index

The first theoretical study about sub-diffraction imaging using metamaterials was done by Pendry [41, 42]. He proposed that a NIM slab with ε = μ = − 1 works as a perfect lens. There had been many debates on whether it is a true perfect lens or not because the slab does not look like a lens; it only works for an object placed on a certain distance and has no focal length [43]. But, NIM slab as a perfect lens was theoretically and experimentally demonstrated in many research groups [24, 25, 41, 44, 45].

**Fig. 3:**
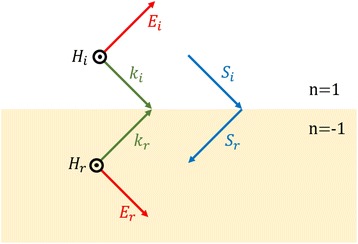
Schematic of negative refraction. Incident ray refracts negatively at the interface of positive index medium and negative index medium. In the negative index material, wavevector $\left(\overrightarrow{\boldsymbol{k}}\right)$ and Poynting vector $\left(\overrightarrow{\boldsymbol{S}}\right)$ have opposite directions in the NIM while they are parallel in the positive index medium

**Fig. 4:**
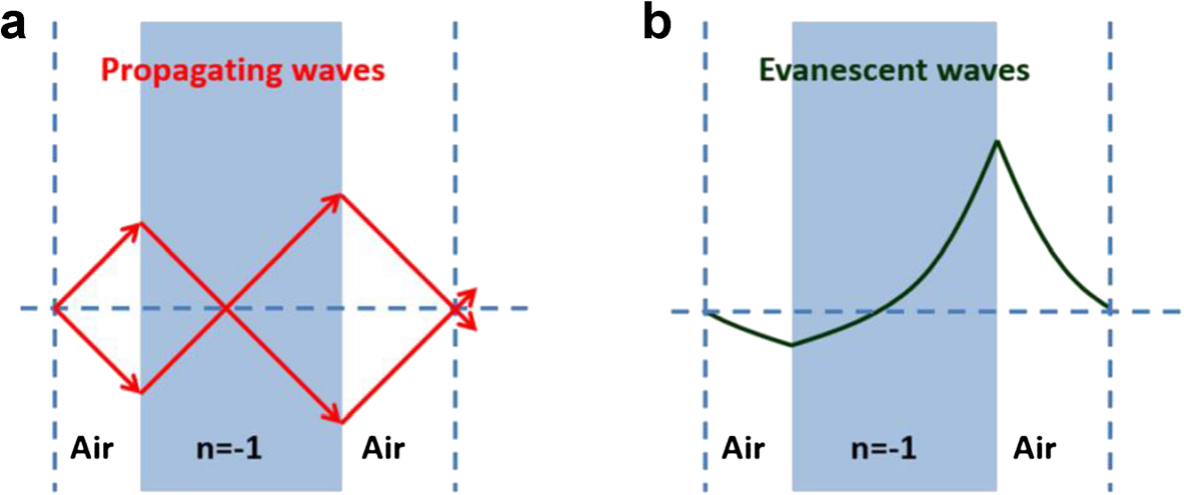
**a** A NIM slab with *n* = − 1 focuses propagating waves from an object to image plane. **b** Amplitude of evanescent waves is enhanced as they pass through the NIM slab

In the negative index medium, energy and phase flow in an opposite direction. Since the wavevector $\overrightarrow{k}$ and the Poynting vector $\overrightarrow{S}$ are anti-parallel, the sign of *k*_∥_ changes at the interface of a positive index and negative index medium as indicated in Fig. 3 from the causality principle and conservation of transverse wavevectors. Evanescent wave which was exponentially decaying is now exponentially increasing in NIMs. In short, evanescent wave is amplified and travels further in NIMs comparing in the positive-index materials, as shown in Fig. 4 (b) [41, 46].

From the reversal of phase and energy flow, phase of propagating wave goes backwards as the ray goes forward, cancelling out phase gained through the same thickness of vacuum. Phase of propagating wave and amplitude of the evanescent wave are restored. Furthermore, all incident light transmits the NIM slab without reflection since the NIM slab and air have identical impedance value. Therefore, a NIM slab that has n = −1 can be considered as a perfect lens in which the image and the object are exactly the same.

Microscopically explaining, amplification of evanescent wave stems from surface resonance [41, 47–50]. When TM waves enter an interface across which the sign of the permittivity is different, oscillation of electron density, called surface plasma oscillation, is generated. If surface mode and the wavevectors of evanescent waves are matched, surface plasmon resonance occurs, generating electromagnetic waves with shorter wavelength that propagate along the interface. These waves, or surface plasmon polaritons (SPPs), have amplitude exponentially decaying along the transverse axis. Since the waves have shorter wavelength than the incident light, light can be tightly confined in a subwavelength size area and the local field intensity can be enhanced significantly. In the process, the evanescent waves are enhanced, leading to sub-diffraction limit imaging.

### Near-field superlens from negative permittivity

The ideal condition to be a perfect lens was ε = μ = − 1, if the dielectric background is air. However, when dealing with very near field or when all dimensions are much smaller than the wavelength of interest, quasistatic limit can be applied. Electric field and magnetic field are decoupled, and the electric and magnetic responses can be considered to be independent. Then under one certain polarization condition, either one of permittivity and permeability negative is sufficient to realize perfect imaging. For TM wave, only negative permittivity (ε < 0, μ > 0) is enough as well [41]. (Note that it is equal to existence condition of SPPs).

**Fig. 5:**
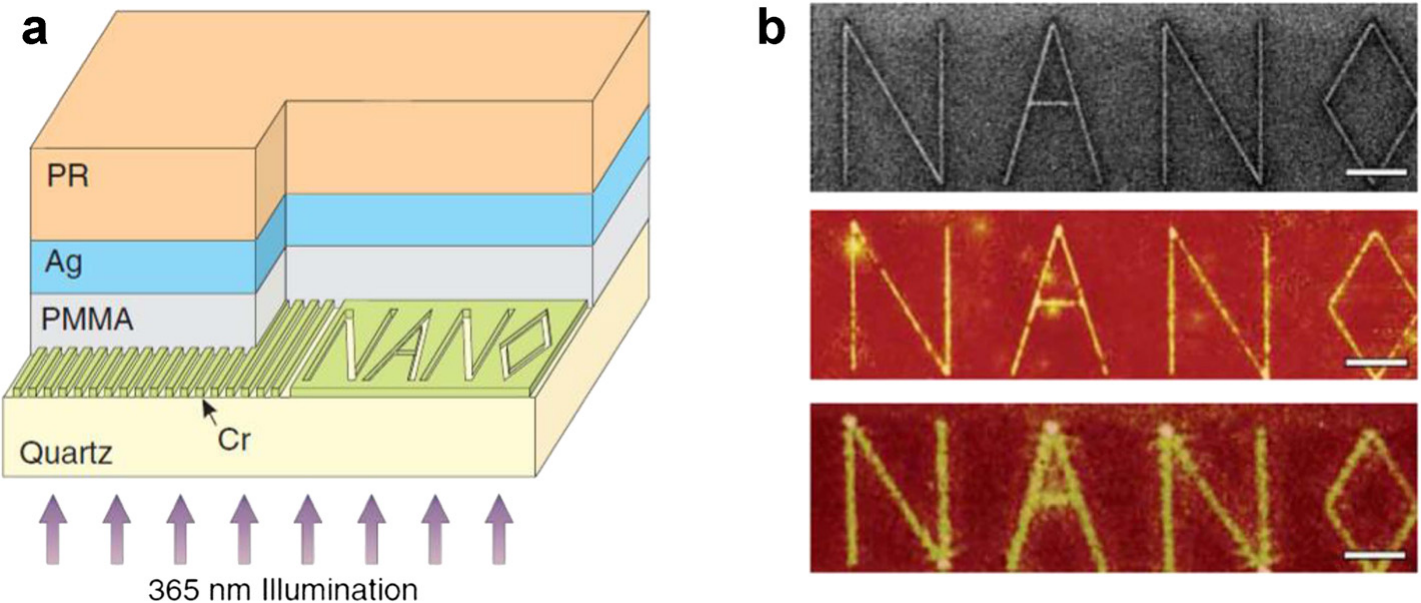
The first experimental demonstration of near-field superlens. **a** Experimental set-up. 60 nm-wide slots of 120 nm pitch and letters ‘NANO’ whose linewidth is 40 nm are inscribed onto chrome. Here, silver slab functions as a superlens. **b** Experimental results. Top: FIB image of the object. Middle: AFM image of the image with the superlens. Bottom: AFM image of the image when the superlens was replaced by PMMA spacer. (The scale bars indicate 2 μm) [52]

From Drude-Lorentz model, metals have negative permittivity at ω < *ω*_*p*_ due to the collective response of free electrons. *ω*_*p*_, a plasma frequency, is normally around ultraviolet (UV) regime for metals. Such a negative permittivity material slab with the thickness much smaller than the wavelength functions as a super-resolution lens, and therefore, is called “superlens” [51]. The first experimental sub-diffraction imaging using superlens was demonstrated in near-field images with 60 nm half-pitch resolution in UV range as shown in Fig. 5 [52].

Though evanescent waves are enhanced, metals’ high resistive losses from the imaginary part of permittivity degrade the image, which is the main limiting factor of the infinite resolution. Thanks to its relatively low losses, silver has been studied as a material for a superlens [41]. Amplification of evanescent waves was numerically [49, 53] and experimentally [48, 49, 53, 54] demonstrated in a thin silver film. Resolution of one-sixth of the incident wavelength at the UV frequency region using silver [52, 55], and resolution of a twentieth of the incident wavelength at mid-IR frequency range using SiC [56] were achieved through numerical simulation and experimental demonstration, respectively. Other designs of superlenses including a silver slab bounded by two different positive index media [47] have been also proposed.

There have been lots of studies to reduce losses in plasmonics materials [57–59]. Use of gain media, materials with negative imaginary part of refractive index, to compensate the losses [60], diluted metals [61, 62], highly doped semiconductors [63, 64], and even superlenses without metal [65] to reduce ohmic-related losses have been proposed since high carrier concentration induces high losses. Nevertheless, high losses were still huge limitations for the practical imaging applications of the superlenses. To minimize losses, the researches to find optimal film thickness and surface roughness requirements that transmit maximum intensity were conducted [42, 49, 52, 66] and moreover, placing multiple thin superlenses to reduce the propagation length in the superlens was proposed [67, 68].

Another limitation of superlenses is polarization dependence. As mentioned earlier, surface plasmons, that are responsible for sub-diffraction imaging, are excited in TM condition. Thus, the superlenses work only for TM wave. Resonance based characteristics also give rise to narrow frequency bandwidth. Working frequency of superlenses has been extended to the mid-IR region [69–71], and multi-wavelength superlens consisting of multilayer of polar dielectric materials exhibiting sub-diffraction imaging in three simultaneous frequencies was also presented [72], while the bandwidths of the individual superlenses are still narrow. Projection type of image with the same size to the object, i.e. no magnification is also one of the limitations. But, the biggest disadvantage is that superlenses work only in the near-field [41, 68]. The distance between the lens and the image plane has to be very short as well as the distance between a source and the lens since evanescent waves decay exponentially again after they passes through the superlens. It prevents the practical super-resolution imaging applications of superlenses.

### Far-field superlens

**Fig. 6:**
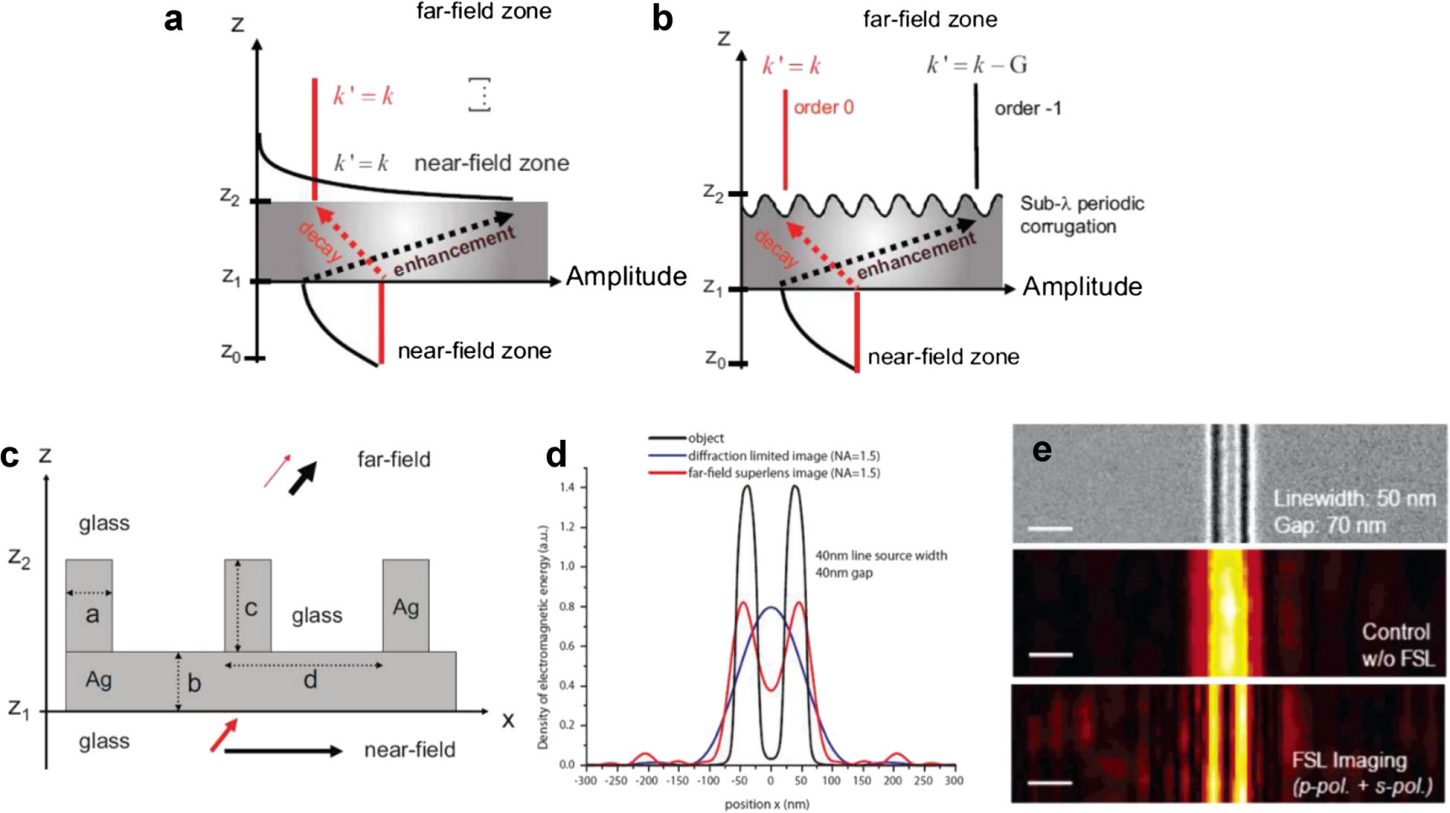
Numerical and experimental results of FSL. **a**, **b** Transmission properties of a conventional superles and a FSL, respectively. Propagating waves and evanescent waves are represented by black and red line, respectively. While the amplitude of the evanescent waves decay exponentially again after they pass through the conventional superlens, evanescent waves are converted into propagating wave at the exit surface of the FSL, making far-field imaging possible [73]. **c** A schematic design of the first far-field superlens. It is a periodically corrugated silver slab in a glass substrate designed for a wavelength equal to 376 nm. a = 45 nm, b = 35 nm, c = 55 nm, d = 150 nm [73]. **d** Simulated electromagnetic density of an object (black) and image plane with (red) and without (blue) the superlens, assuming numerical aperture (NA) is 1.5. Here, an object is composed of two 40 nm lines separated by 40 nm [73]. **e** Experimental demonstration of the far-field superlens, where an object is a pair of wires with line width of 50 nm and spacing of 70 nm. NA = 1.4, and the wavelength is 377 nm. Top: SEM image of an object. Middle: image without superlens. Bottom: reconstructed FSL image. (Scale bars indicate 200 nm) [75]

To overcome the limitation arisen in the near-field superlens, the efforts to project the sub-diffraction image onto the far-field have been made. In 2006, a far-field superlens (FSL), a periodically corrugated planar superlens, was proposed (Fig. 6 (c)) [73]. Studies about transmission properties of the periodic grating as waves pass through the FSL were conducted [73–76]. It was numerically demonstrated that the FSL can resolve 40 nm lines with a 30 nm gap at 376 nm wavelength (shown in Fig. 6 (d)) [73]. Also, 50 nm lines separated by 70 nm (shown in Fig. 6 (e)) [75] and 50 nm features separated by 20 nm [76] were resolved experimentally at 377 nm wavelength.

For the far-field sub-diffraction imaging, evanescent waves have to be not only amplified, but also converted into propagating waves. It is fairly similar to the near-field superlens except that there are subwavelength scale periodic metallic gratings on the exit surface. It was proven that under TM condition, a periodic metallic corrugation on the lens surface couples evanescent waves and propagating waves [73].

As the evanescent waves from the object transmit through a nanopatterned superlens, the near-field image and the grating pattern are overlapped, generating a moiré pattern, an interference pattern created when two periodic or quasi-periodic patterns are superimposed [77, 78]. The spatial frequency of the moiré pattern is equal to the difference of the frequency of the evanescent wave and the grating pattern. Even if either the evanescent waves or the grating is not resolvable due to diffraction limit, the moiré pattern can be resolved if it has lower wavevector than the spatial resolution. Therefore, the near-field image can be numerically reconstructed by analyzing moiré fringes as long as the grating pattern is known [79]. Since period of the grating pattern is limited only by fabrication, not by diffraction, super-resolution which had been impossible before was realized [76].

More generally, the near-field and far-field angular spectrum has a one-to-one relationship, following the grating law. Transverse wavevectors of transmitted propagating waves (*k* ^'^) can be expressed in terms of transverse wavevectosr of the incident evanescent wave (*k*) and the grating wave number (*G*): *k* ^'^ = *k* + *pG*, where *p* is the diffraction order [73]. When *p* is negative and the grating wavenumber is large enough, the wavevectors of the transmitted waves is reduced enough to propagate, resulting in far-field sub-diffraction imaging. Transmission of sub-diffraction image through near-field superlens and far-field superlens when the diffraction order is equal to −1 are illustrated in Fig. 6 (a) and (b), respectively [73].

The image, obtained as a field angular spectrum, has to be transformed into a real space image via inverse Fourier transformation. It makes real time imaging impossible, which is the main obstacle of FSL imaging [73]. To achieve real time imaging, a FSL with a different mechanism which functions for a certain range of wavevectors was investigated [74, 75]. Also, the earlier researches have focused on UV region, but the wavelength range was expanded to visible frequencies by changing permittivity of the materials [80].

By investigating absorption and transmission factor for TM and TE waves, another research revealed that the conversion of evanescent waves into propagating waves in FSL results from excitation of SPPs supported by the metallic grating. Incident TM waves scattered at the exit surface excite SPP modes, giving rise to large absorption factor [74]. Meanwhile, it was announced that SPPs play a negative role in *p* = 0 transmission [81]. From these two facts, evanescent waves in the negative diffraction order are strongly transmitted compared to propagating waves in the order 0, enabling sub-diffraction imaging.

### Hyperlens

Another metamaterial-based lens enabling far-field sub-diffraction imaging is hyperlens. Hyperlenses are made of a hyperbolic metamaterial, a highly anisotropic material with hyperbolic dispersion. These materials have different signs of permittivity with regard to the directions. That is to say that permittivity in the parallel direction and perpendicular direction to the anisotropic axis has different sign. Such materials with anisotropic permittivity tensor act like metals in one direction and dielectrics in the other direction. Accordingly, from the dispersion relations in anisotropic medium, hyperbolic metamaterials have hyperboloid isofrequency surface, as can be seen in Fig. 1 (b) and (c). Due to this abnormal property, there have been many researches and it was discovered that hyperbolic metamaterials can realize enhancement in spontaneous emission, very large photonic density of states [38, 82–85], thermal engineering [86–88], and of course sub-diffraction imaging.

**Fig. 7:**
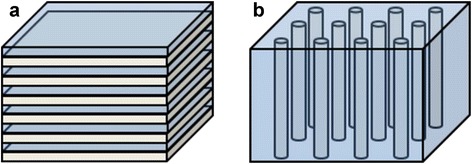
Hyperbolic metamaterial structures. **a** metal-dielectric alternating multilayer, **b** nanowire array

Hyperbolic metamaterials generally can be realized by two types of structures. As shown in Fig. 7 (a) and (b) respectively, metal and dielectric are stacked in a subwavelength scale alternating multilayer, and parallel sub-wavelength sized metallic wire array are embedded in a dielectric template.

When light passes through such structures, SPPs propagate along the metal-dielectric interface, decaying exponentially along the direction perpendicular to the propagation. Since all dimensions are much smaller compared to the wavelength, SPPs of the different layers interact with each other and cause a collective response. Therefore, we can consider hyperbolic metamaterials as homogeneous media and apply an effective medium approximation. For example, effective permittivity of metal-dielectric multilayer structure follows the equation (2).

2 $$ {\varepsilon}_{\perp }=\frac{\varepsilon_m{d}_m+{\varepsilon}_d{d}_d}{d_m+{d}_d},\kern0.5em \frac{1}{\varepsilon_{\parallel }}=\frac{d_m/{\varepsilon}_m+{d}_d/{\varepsilon}_d}{d_m+{d}_d} $$

Here, *d* indicates thickness of each structure, the subscripts ⊥ and ∥ denote components perpendicular and parallel to the anisotropy axis, and the subscripts *m* and *d* indicate metal and dielectric material, respectively. Effective permittivity *ε*_∥_ and *ε*_⊥_ can be represented as functions of permittivity and thickness of metal and dielectric material. By tuning these parameters properly, hyperbolic dispersion can be achieved.

**Fig. 8:**
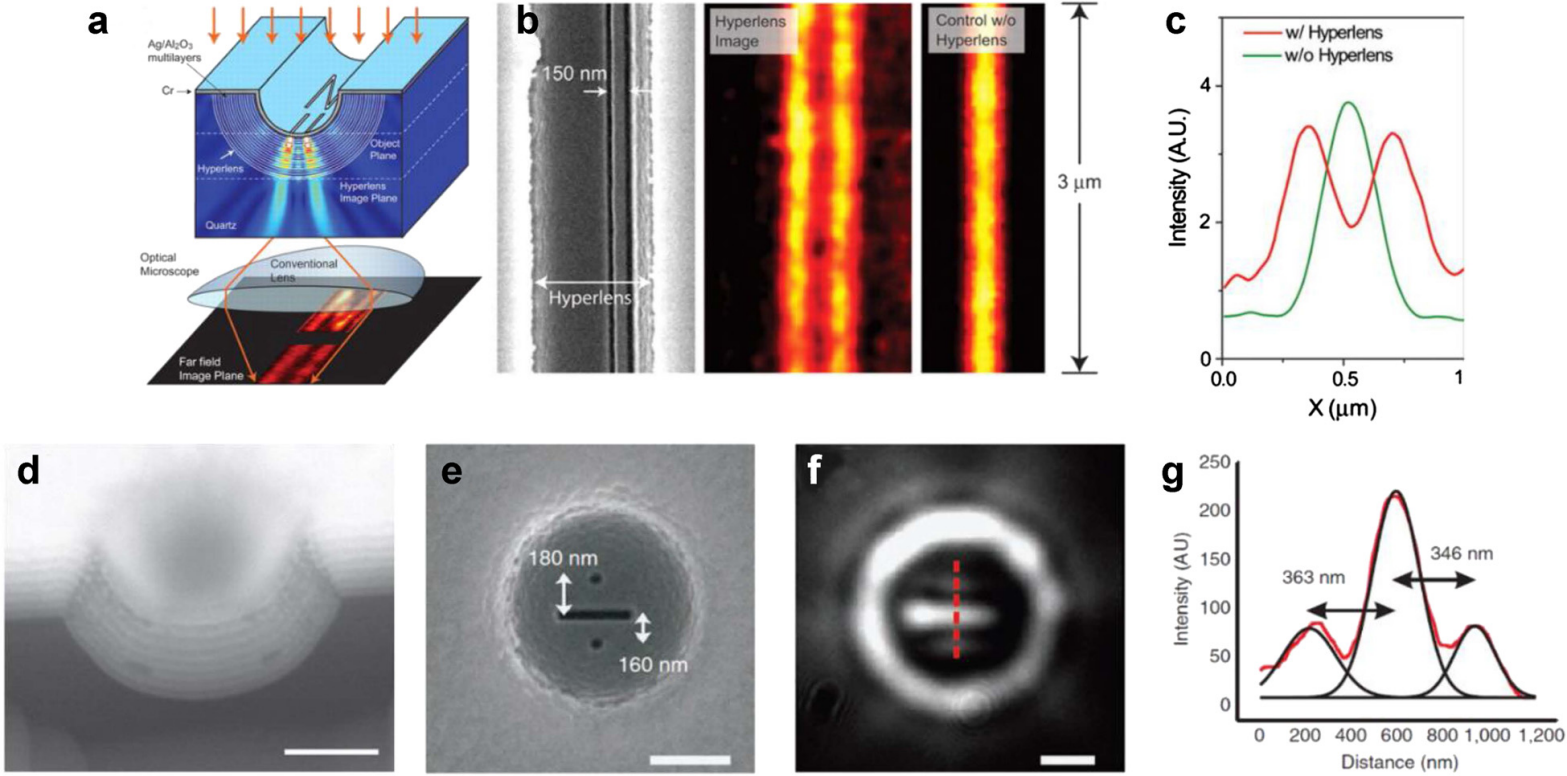
**a** Schematic of a super-resolution imaging using cylindrical hyperlens [92]. **b** Experimental demonstration of the hyperlens. Two 35 nm lines are separated by 150 nm, and the wavelength of the incident light is 365 nm. From left to right, SEM image of the object, image with the hyperlens, and image without the hyperlens, respectively [92]. **c** Average intensity of the cross section of the line image shown in (**b**). Red and green indicate average intensity with and without the hyperlens, respectively. (A.U. means arbitrary units.) [92] **d** SEM image of the cross-sectional view of spherical hyperlens. (Scale bar indicates 500 nm) [94]. **e** SEM image of an object. **f** Magnified image obtained by using spherical hyperlens. **g** Red line shows average intensity of the cross section of the obtained image shown in (**f**). (All scale bars indicate 500 nm) [94]

A theoretical concept of hyperlens was proposed by different groups in 2006 [89–91]. It has metal-dielectric multilayer structure in a cylindrical geometry so that sub-diffraction image can be transferred and magnified. Sub-diffraction imaging using hyperlens was experimentally demonstrated in UV [92, 93] and visible [94, 95] frequency range. 130 nm spatial resolution and magnification of two were demonstrated by using cylindrical shape hyperlens in 365 nm wavelength range [92, 93]. Detailed information regarding cylindrical and spherical hyperlens is shown in Fig. 8.

Hyperlens with tapered metallic nanowire array has been also studied numerically and experimentally [96–100], and resolution of one-fifteenth of a wavelength and magnification ratio of three were achieved numerically [99]. In this nanowires structure, sub-diffraction imaging is based on the excitation of the longitudinal resonance mode of the individual rods. Meanwhile, the sub-diffraction image reconstruction using multilayered hyperlenses relies on the excitation of the transverse resonance modes of SPPs. To understand the mechanism of hyperlens more specifically, let’s consider a cylindrical hyperlens with *ε*_*r*_ < 0, *ε*_*θ*_ > 0. Since it has an open isofrequency surface, arbitrarily large *k*_*θ*_ with real *k*_*r*_ is allowed in the lens. When evanescent waves enter the hyperlens, propagating modes of the lens are excited. In other words, evanescent waves are converted into propagating waves inside the hyperlens. During the propagation, *k*_*θ*_ *r* is a constant along the radial direction from the angular momentum conservation. *k*_*θ*_ decreases as the wave propagates, so the sub-diffraction limited image is magnified by the factor of the outer radius divided by the inner radius. If *k*_*θ*_ is compressed enough at the outer boundary so that *k*_*r*_ becomes real value, evanescent waves becomes propagating waves at the outside of the hyperlens, enabling far-field propagation of the sub-diffraction information. Integrating with the conventional wide-field microscopes, the magnified resolution which is no more diffraction-limited can be captured by the regular objective lenses and CCD-cameras in real-time. Such benefits make hyperlenses more promising for the practical imaging solution.

In the cylindrical geometry proposed at the first time, super-resolution was allowed in only one direction and TM mode. Later, two full lateral dimensional sub-diffraction resolution was achieved by spherical shape of hyperlens with the unpolarized light at visible frequency, as shown in Fig. 8 (d) to (g) [94]. However, because both cylindrical and spherical geometry are not easy to position the object into the curved geometries, the concept of flat hyperlens was proposed. Hyperlens embedded in a metamaterial slab [101] and planar lenses using hyperbolic dispersion from metal-dielectric multilayered structure [102] was proposed as a solution. A flat hyperbolic metamaterial to which subwavelength grating is added was proved to be capable of far-field super-resolution imaging at visible frequency [103, 104].

Although a hyperlens provides far-field imaging, there are still some limitations. For example, an object has to be placed very close to the inner surface of the lens just like at the superlens. Hyperlens also suffers from high losses like superlens. Many researchers have tried to find hyperbolic metamaterial with lower losses, and as results, suggested to use active media [105, 106] and transparent conducting oxides (TCOs) to replace metals in near-IR range. Hyperbolic metamaterials composed of TCOs and dielectric exhibited low losses compared to the conventional metal-based ones [107, 108]. As a hyperlens becomes thicker, the image is more degraded due to the high absorption losses. However, a research proposed a hyperlens capable of sub-diffraction imaging at visible frequency with thickness as large as required. Using canalization, resolution of one-twentieth of the wavelength was confirmed [109].

For the high quality image, group velocity should remain a constant during the propagation. Since the group velocity is orthogonal to the dispersion curve, hyperlens with flat hyperbolic dispersion curve, which can be achieved by large negative *ε*_*r*_ and small positive *ε*_*θ*_, is better. Another requirement of the undistorted image is an impedance matching condition, which indicates that a metal and a dielectric material should have similar order of polarization response. Since metals have real part of permittivity one order higher than that of dielectric materials, fill fraction of the metals has to be much smaller. It became a fabrication challenge, because grain structure of the thin film metal causes grain-boundary scattering for free electrons, lowering the image fidelity [110, 111]. Other factors responsible for losses are large reflection at the surface and scattering through the lens [112]. To reduce the surface reflection, studies about impedance-matched hyperlens was dedicated [113].

While hyperbolic metamaterials we have discussed so far are requiring difficult fabrication and suffering from high loss, hexagonal boron nitride, a natural hyperbolic material, has been emerged as a great alternative recently. Hyperbolic polaritons confined in a sub-diffractional volume were observed in a crystal of hexagonal boron nitride [114] and the sub-diffraction imaging lenses in IR frequency range were demonstrated [114–116]. Also, as alternatives to the hyperbolic materials with highly eccentric elliptic dispersion or extremely large refractive index (negative index is not necessary) works as well as hyperbolic metamaterials despite of the cut-off frequency in elliptic dispersion since the goal is supporting large *k*_*θ*_ [117].

### Metalens

**Fig. 9:**
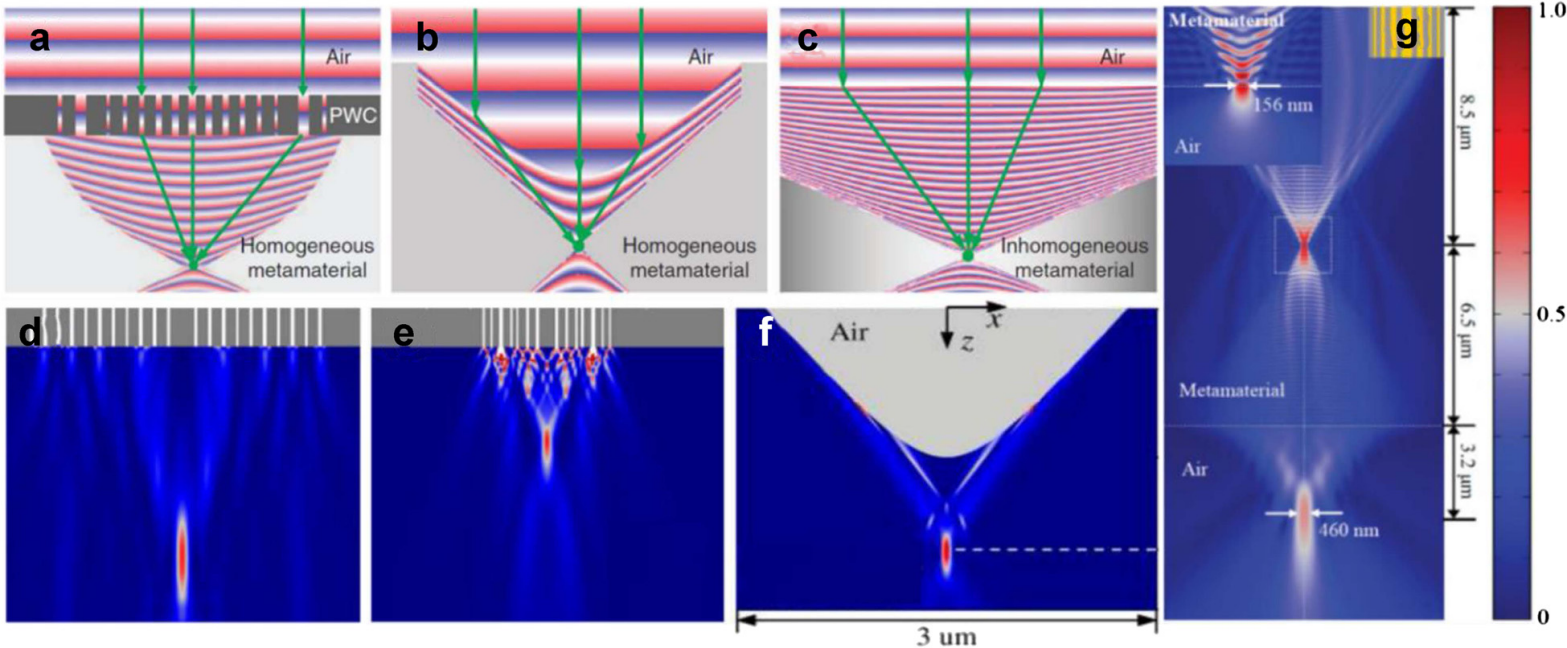
Numerical simulation of three types of metalenses. Schematics of (**a**) plasmonic waveguide coupler (PWC) metalens (**b**) metamaterial immersion lens (MIL) and (**c**) hyperbolic gradient-index (GRIN) metalens [121]. A normal plane wave is focused by using metalens composed of an Ag PWC and (**d**) an elliptical dispersion material, and (**e**) a hyperbolic metamaterial [118]. **f** Focusing of a normal incident plane wave using a MIL. Permittivity in the direction parallel to the incident is negative, while permittivity in the lateral direction is positive [119]. **g** Focusing of a normal incident plane wave using hyperbolic GRIN metalens. Electrical intensity distribution is represented by colors. The left inset shows the zoom-in focus area when the metalens is truncated at its internal focal plane [120]

Lenses discussed so far (perfect lens, superlens and hyperlens) have a limitation in common; incapability to focus a plane wave into a spot. There is no phase compensation mechanism, which supports a plane wave focusing and Fourier transform function. As alternative approaches to achieve them, three types of metalenses capable of both sub-diffraction imaging and Fourier imaging were suggested by different groups (Fig. 9) [118–121]. Plane wave focusing is available by introducing a phase compensation mechanism and a coupling between the metamaterial and air. Hence, the metalenses can be integrated with conventional microscope easily.

The first one consists of a plasmonic metal-insulator-metal waveguide coupler (PWC) and a hyperbolic (or highly eccentric elliptic) metamaterial slab [118]. A plane wave can be focused to a point as it passes through a PWC and a metamaterial slab in order. When the incident plane wave excites the PWC, the waves with different transverse wavevectors and different phase transmit the metamaterial slab. By varying geometric parameters (width and height of the PWC), PWC can be designed so that the sum of phase gain from the metamaterial slab and from the PWC is a constant for every path. Under such a condition, plane waves interfere constructively at a point without loss of high spatial frequency waves thanks to unusual dispersion. Using this phase compensation mechanism, plane wave focusing and magnification are possible. Inversely, radiated beams from a point source are converted into a plane wave as they go through a metamaterial slab and a PWC. Without the PWC, a total internal reflection occurs when the waves with high wavevectors pass the exit surface of the lens, inhibiting the transmission of evanescent waves. But here, the PWC takes a role of bidirectional wavevector coupling and phase matching. The validity of the PWC-based metalens was demonstrated numerically [118].

The second type of metalens is metamaterial immersion lens (MIL) [119]. MILs have same phase compensation mechanism with a conventional lens; geometric shape of a MIL determines propagation length and hence, phase accumulation. Since diffraction phenomenon limits resolution to $\frac{\uplambda}{2\mathrm{N}\mathrm{A}}$ where NA is a numerical aperture, it is important to make a lens with high NA. To achieve high NA, properly shaped high-index metamaterials were introduced. Interface of a MIL have to be designed to realize bidirectional coupling between the lens and air. By using a concave-shaped hyperbolic MIL where real part of permittivity along the propagating direction is negative, plane wave focusing was demonstrated. Whereas a convex shape is used to focus a plane wave for conventional materials, the hyperbolic MIL has concave surface due to negative refraction at the metamaterial-air interface [119]. One interesting thing is that the losses are important factors in the working mechanisms of MIL metalens. The waves with different transverse wavevectors suffer different amount of the loss due to their different loss coefficients, propagation length, and refraction angle. In a MIL, the waves with high transverse wavevectors attenuate less, and therefore, contribute more to construct an image, leading to high NA [119].

In a MIL, since the geometry is responsible for beam bending, the image quality is restricted by the geometrical and wave aberration. However, the conventional imaging devices with non-flat geometry can be compressed to the flat one using coordinate transformation because metamaterials are able to control light in almost arbitrary manners [122]. A flat metamaterial with gradually varying refractive index was proposed as an alternative metalens [120]. In the third metalens, a gradient-index (GRIN) metalens, spatially varying refractive index plays a role in bending light. A GRIN metalens is quite similar to a conventional GRIN lens, but the difference is that refractive profile is complicated in GRIN metalens while it is symmetric in the conventional one. A metalens consisting of patterned copper strips and an FR4 as a matrix in which refractive index is addressed by changing a proportion of the copper strips was proposed [123]. Since the copper strips are not resonant in the operation frequency, this metalens, or flattened Luneburg lens, exhibited low losses and broadband operation. Because artificial magnetic response is difficult to be fabricated, GRIN metalens in which permittivity distribution is designed in order to focus a plane wave was introduced and achieved a resolution of one-sixth of a wavelength [120]. A resolution of one-hundredth of a wavelength, and either wavelength- or incident angle- dependent focusing was numerically achieved by using metallic waveguide on which subwavelength patterns are non-periodically arranged [124]. A switchable GRIN metalens by shorting the gaps in SRRs were also presented [125].

Furthermore, other types of metalenses from the transformation optics approach have been proposed [126–129]. Planar metamaterial lenses which are capable of both plane waves focusing and magnifying sub-diffraction-limited objects have been studied [101, 122, 128, 129]. Restricted operating conditions of the planar metalens such as narrow bandwidth and directions of incident light were investigated and expanded by further research [130], but still remain as the major limitations.

### Flat lens based on a metasurface

High losses, high fabrication cost, and complicated fabrication process have been pointed out as the main problems of plasmonic metamaterials [131, 132]. Plasmonic metamaterials with very thin thickness compared with the wavelength of the incident light are free from those disadvantages. These ultrathin planar metamaterials, called metasurfaces, can be also easily integrated with conventional optical devices due to their planar geometry.

While the convergence of optical waves relies on a gradual phase accumulation during the propagation in conventional lenses, focusing mechanism resulting from abrupt phase change can be achieved using phased array metasurfaces. Suppose meta-atoms, unit cells of a metasurface, are separated by a sub-wavelength distance and the interaction of the near-field is relatively small. When an oscillating electric field is applied to the metasurface, free electrons in meta-atoms oscillate forced by the electric field and restoring force. The strong interaction between incident light and the localized surface plasmons of meta-atoms is the key of the phase shift. Meta-atoms with spatially varying geometry work as resonators, giving rise to phase discontinuity through the sub-wavelength thickness [133, 134].

Even though metasurface is a relatively new concept, flat plates with nano-arrays to control phase gradient have been investigated in radiofrequency region since 1990s [135–138]. Phase shift has been realized using reflectarray, transmitarray, and array of nanoholes, optical masks, or nanoslits in optical frequency range [137–143]. Relationship between the phase gradient, reflection angle and refraction angle follows generalized Snell’s law [144].

3 $$ \sin {\theta}_r- \sin {\theta}_i={n_i}^{-1}{k_0}^{-1}\nabla \Phi $$

4 $$ {n}_t \sin {\theta}_t-{n}_i \sin {\theta}_i={k_0}^{-1}\nabla \Phi $$

**Fig. 10:**
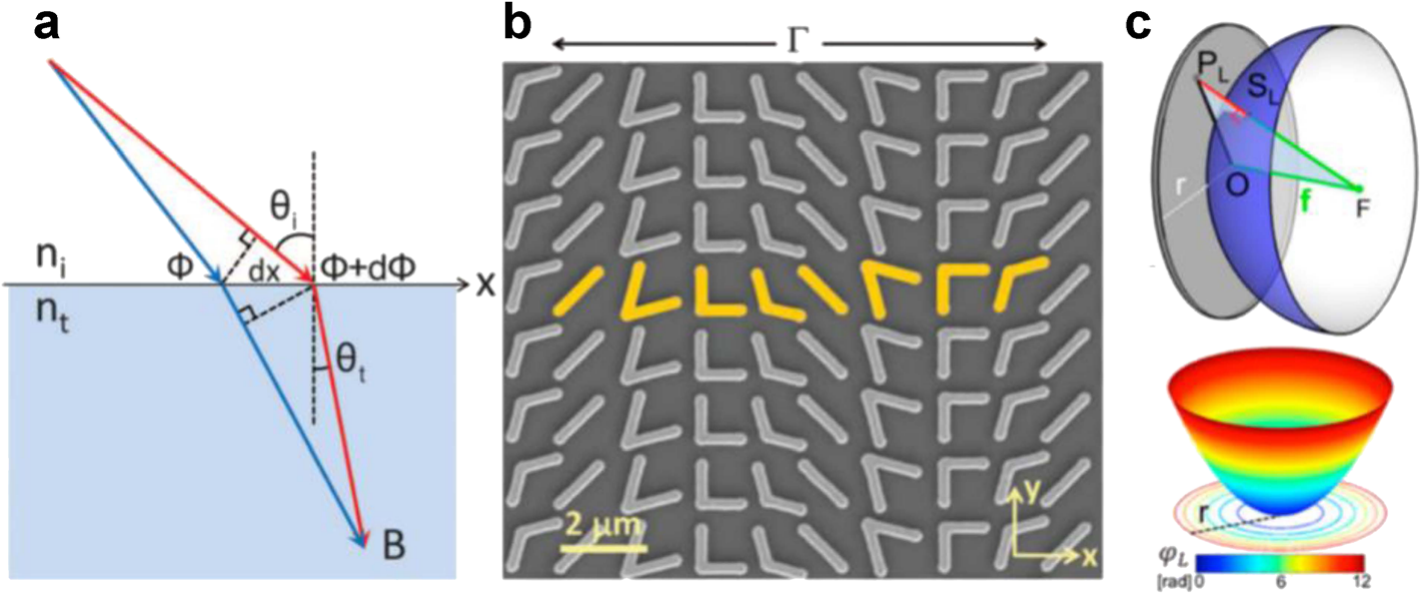
**a** A schematic diagram which helps the understanding of the generalized Snell’s law of refraction. Refraction angle is affected by an additional phase discontinuity term. [144] **b** SEM image of nanoantenna array fabricated on a silicon wafer. The unit cell represented as a yellow color is periodically arranged with a periodicity of Γ. Each V-shaped antennas have 220 nm width and 50 nm thickness [144]. **c** Top: schematic of a flat lens. Bottom: phase distribution on the flat lens. Hyperboloidal radial phase distribution makes an incident plane wave focus on a point F [145]

*θ*_*i*_, *θ*_*t*_, and *θ*_*r*_ are the angles of incidence, refraction, and reflection, respectively, *n*_*i*_ and *n*_*t*_ are the refractive indices of the two media on the incidence and transmission side, respectively, *k*_0_ is a free-space wave number space, and ∇Φ is the gradient of phase along the interface. Equation (3) is for reflection and equation (4) is for refraction. The generalized Snell’s law, which is basically a momentum conservation equation, indicates that the direction of the refracted or reflected light (in this case, refracted light) can be controlled by assigning a proper phase gradient across the metasurface (Fig. 10 (a)). Namely, the phase-gradient metasurface can be designed to have a desired phase shift distribution by arranging properly-designed meta-atoms.

In an ideal phase-gradient metasurface, phase discontinuity strongly depends on the wavelength of incidence and geometry of the meta-atom, spanning all range from 0 to 2π. Beams that reach different position in metasurface refract with different angle as they pass through the metasurface in which meta-atoms with different geometry are arranged. When the image is reconstructed at a certain point by constructive interference of the refracted light, the metasurface functions as a lens. Since metasurfaces can control wavefront in nearly arbitrary manners, metasurfaces can be used in many applications. For example, they have been used to generate holograms, Gaussian beam, Bessel beams and optical vortex [144–147], and many researchers have shown that phase, amplitude, polarization state, frequency, momentum and angular momentum can be manipulated using metasurfaces [146, 148–150]. However, only metasurfaces related to imaging will be discussed in this review. It is also noted that the fundamental principles of the phase-gradient metasurface, which are related to the Pancharatnam-Berry phase [151], will not be discussed here.

The first demonstrated phase-gradient metasurface is shown in Fig. 10 (b) [144]. A unit cell of this nanoantenna-array (NA) metasurface is composed of eight symmetry breaking V-shaped nanoantennas with subwavelength size and separation. When an electromagnetic wave is applied, these V-antennas support two modes: symmetric modes and antisymmetric modes. Along the x-direction, two modes are excited with different phase and amplitude, resulting from the linear variation of the angle between the two rods of the antenna. The antennas with different geometry give rise to different phase shift. One-dimensional linear phase discontinuity results from the periodically patterned V-antennas. NA metasurface where nanoatennas are made of gold was analytically proved to enable 2π range of phase shift for cross-polarized light. [144] Not only V-shaped, but U- or C-shaped antennas were also used [152]. Modulation of phase, amplitude and polarization arising from two modes supported by V- and Y-shaped antennas was studied theoretically and experimentally [153].

Using phase shifting ability, planar lenses based on NA metasurface were presented. Aberration-free focusing using gold metasurface was experimentally demonstrated at THz region. Thin gold film on which V-shaped nanoantennas are radially distributed has hyperboloidal phase profile so that the scattered waves interfere constructively at the focal plane. These flat lenses exhibited high NA as well as zero monochromatic-aberration [145]. After that, metasurface-based lenses with complementary nanoantennas were proposed. A metasurface composed of array of complementary V-shaped antennas was fabricated in a gold film using photolithography and showed reduced noise due to directly transmitted light [154]. Another experimental research demonstrated a strong focusing ability of concentrically perforated gold films called a Babinet-inverted metalens. This metasurface accomplished a focal length of a few nanometers, high signal to noise ratio, and wavelength-controllable focal length by replacing plasmonic nano-antennas to nano-voids [155].

The phase shifting ability can be used to surpass the chromatic aberration, which is caused because light with different wavelength has different refractive angle. Recently, a metasurface based on dielectric resonators showed wavelength-dependent phase shift so that waves with three different wavelength deflect into the same angle [156]. In addition, metasurfaces acting as a lens in which either the incoming light converges or diverges depends on the helicity of the incidence were presented [157, 158]. When circularly polarized light enters the metasurfaces, the polarity is determined by the polarization of the light while the polarity of the conventional lenses are not interchangeable and thus, both magnification and demagnification are possible in the metasurface.

Although metasurfaces have showed new possibilities for imaging and further, manipulation and control of electromagnetic waves, they do not provide sub-diffraction imaging yet. Overcoming diffraction limit in imaging using flat lens based on metasurfaces remains as a future work.

### Other lenses

Remarkable properties of metamaterials can be also extended to engineer acoustic waves, magnetic fields and thermal energy, as well as imaging optical waves.

1. Acoustic metamaterial lens

**Fig. 11:**
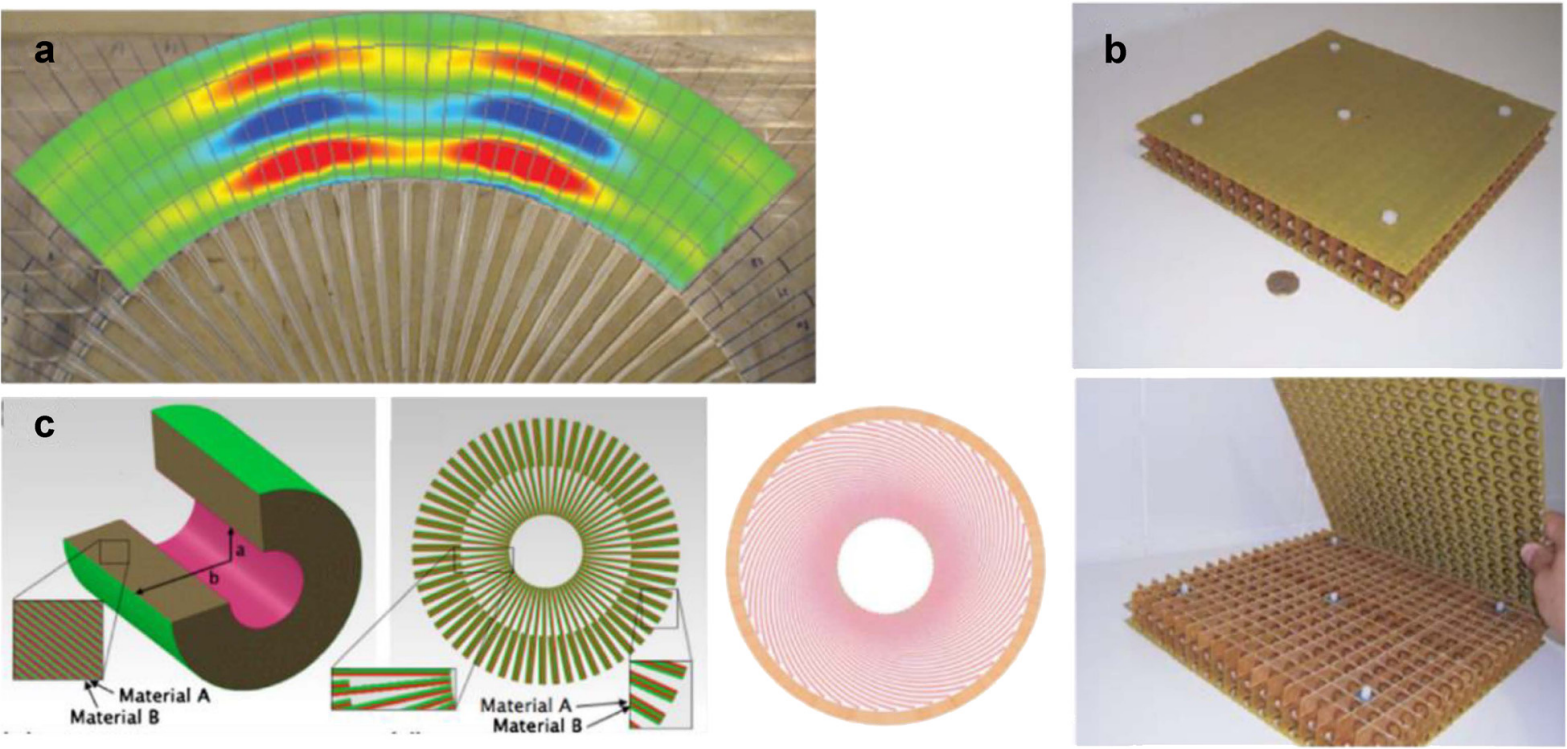
**a** Experimental pressure measurements of an acoustic hyperlens consisting of 36 brass fins [166]. **b** Images of the magnetic metamaterial lens composed of 3D array of capacitively loaded rings [167]. **c** Thermal metamaterial lens. From left to right, thermal shield, cross-sectional schematic for thermal concentrator, and cross-sectional schematic for heat flux inverter [170].

Diffraction-free acoustic imaging using metamaterials allows more efficient underwater sonar sensing, medical ultra-sound imaging, and non-destructive materials testing. Focusing acoustic waves using phononic crystals [159, 160] and metamaterials [161–163] has been studied. An acoustic lens consisting of double C-shaped voids in thin stiff sheets [164] and a magnifying acoustic hyperlens where materials with positive and negative dynamic density are stacked alternatively [165] were proposed. Since those lenses involved a resonance, narrow bandwidth and high losses became problematic. Far-field sub-diffraction imaging of acoustic waves in a non-resonant acoustic magnifying hyperlens composed of 36 brass fins in air embedded on a brass substrate was experimentally demonstrated. Strictly, this hyperlens does not have a hyperbolic dispersion relation, but has an elliptic one. Since imaging is not affected by a resonance, high propagation distance and magnification ratio can be achieved (Fig. 11 (a)) [166].

2) Magnetic metamaterial lens

As mentioned before, quasistatic limit can be applied in sub-wavelength scale structures. Since artificial magnetism in frequency range above 1 THz is challenging, focusing using superlenses and hyperlenses have been usually achieved by negative permittivity. However, in some applications, metamaterial lens with negative magnetic response is more preferred. For instance, in radio frequency regime, where the wavelength is the order of meters, designing and fabricating negative permeability materials are relatively easy. A metamaterial lens with *μ*_*r*_ = − 1 using surface coils can focus the radio frequency magnetic field, and therefore can be used in many applications such as magnetic resonance imaging (Fig. 11 (b)) [167] or wireless power transfer [168].

3) Thermal metamaterial lens

As optical metamaterials transfer sub-diffraction optical information, the manipulation of nanoscale heat flux was realized by using metamaterials. It originates from the surface modes coupling which gives rise to a large near-field heat transfer modulation [169]. Furthermore, a multilayered composite material was proved to have abilities to shield, concentrate, and invert heat flux from its anisotropic characteristic (Fig. 11 (c)) [170]. Hyperbolic metasurfaces, very thin hyperbolic metamaterials, can also be used to control thermal radiation and near-field heat transfer, thanks to the diverging photonic density of state. Hence, controlling heat flow becomes possible, resulting in various applications including imaging, sensing, and detection [171].

## Conclusion

In this review, we discussed how metamaterials have been used for better imaging surpassing and complementing the currently available optical microscopes. Metamaterials have shown ability to control electromagnetic wave at the nanoscale, allowing nanometers order of imaging resolution to come true. Over the past few decades, metamaterials with various designs and structures have been investigated for super-resolution imaging field to offer many different types of lenses which can overcome diffraction limit.

There are, however, some practical problems that hold real life applications. Although there have been intensive researches and tremendous efforts to achieve new science and technology, the major limitations of metamaterials – high losses, deep nanoscale fabrication issues, and narrow range of frequency, angle and polarization dependency, materials and size flexibilities, and integration with the conventional optics components- are still remained as future works. However, it is for sure that metamaterials have showed tremendous possibility and potential to change a paradigm in many fields, especially in optical imaging we discussed here. Therefore, with the development of nanofabrication and nanomanufacturing methods, and the integration of new creative ideas, overcoming the results and limitations mentioned in this review will be the continuous efforts to make metamaterial-based imaging techniques to be a next generation of imaging technology replacing current optical microscopy, which thus can be called nanoscopy.
